# HN1 as a diagnostic and prognostic biomarker for liver cancer

**DOI:** 10.1042/BSR20200316

**Published:** 2020-07-30

**Authors:** Zhicheng Liu, Dingquan Yang, Yanqing Li, Yan Jiao, Guangchao Lv

**Affiliations:** 1 Department of Gastrointestinal Surgery, The First Hospital of Jilin University, Changchun, Jilin 130021, P.R. China; 2 Department of Gastrointestinal and Colorectal Surgery, China-Japan Union Hospital, Jilin University, Changchun, Jilin 130033, P.R. China; 3 Department of Pathophysiology, College of Basic Medical Sciences, Jilin University, Changchun, Jilin 130021, P.R. China; 4 Department of Hepatobiliary and Pancreatic Surgery, The First Hospital of Jilin University, Changchun, Jilin 130021, P.R. China; 5 Department of Thoracic Surgery, The First Hospital of Jilin University, Changchun, Jilin 130021, P.R. China

**Keywords:** diagnosis, Hematological and neurological expressed 1, HN1, liver cancer, prognosis

## Abstract

**Background:** The present study aimed to examine the diagnostic and prognostic value of HN1 in terms of overall survival (OS) and recurrence-free survival (RFS) in liver cancer and its potential regulatory signaling pathway.

**Methods:** We obtained clinical data and HN1 RNA-seq expression data of liver cancer patients from The Cancer Genome Atlas database, and analyzed the differences and clinical association of HN1 expression in different clinical features. We uesd receiver-operating characteristic curve to evaluate the diagnosis capability of HN1. We analyzed and evaluated the prognostic significance of HN1 by Kaplan–Meier curves and Cox analysis. Gene Set Enrichment Analysis (GSEA) was used to identify signaling pathways related to HN1 expression.

**Results:** HN1 mRNA was up-regulated in liver cancer, and was associated with age, histologic grade, stage, T classification, M classification, and vital status. HN1 mRNA had ideal specificity and sensitivity for the diagnosis (AUC = 0.855). Besides, the analysis of Kaplan–Meier curves and Cox model showed that HN1 mRNA was strongly associated with the overall survival and could be well-predicted liver cancer prognosis, as an independent prognostic variable. GSEA analysis identified three signaling pathways that were enriched in the presence of high HN1 expression.

**Conclusion:** HN1 serves as a biomarker of diagnosis and prognosis in liver cancer.

## Introduction

Liver cancer is one of the malignancies with a high mortality rate. According to the latest statistics of 2018, 782,000 died of liver cancer, and the cancer mortality rate ranks fourth in the world [1]. Despite diagnostic techniques and the treatment means to become more abundant for liver cancer [2–5], the improvement of liver cancer prognosis is still disappointing [6]. Recently, exploring specific biomarkers and integrating them into clinical prognosis evaluation have become one of the main research directions of cancer [7].

Hematological and neurological expressed 1 (HN1) also known as Jupiter microtubule-associated homolog 1 (JPT1) was first discovered in mouse embryos and is located on human chromosome 17q25.2 [8,9]. Although the functional role of HN1 is still not completely clear in human cells, current studies indicate that HN1 is involved in regulating cell cycle, growth, repair, and regeneration processes in embryo, retinal, hematopoietic and neurologic cells, and the high conservation of this gene suggests its function is important to the human [10–12]. In the early time, Huang et al. found that HN1 was up-regulated and associated with cancer metastasis in prostate cancer [13]. Then HN1 was found to be highly expressed in several cancers, such as lung cancer [14], breast cancer [15,16], melanoma [17], malignant gliomas [10], and epithelial ovarian cancer [18].

However, no studies investigated the association between HN1 expression and liver cancer up to now. In the present study, we explored the relationship between HN1 mRNA and clinical features of liver cancer and evaluated the potential value of HN1 mRNA in the diagnosis and prognosis of liver cancer patients.

## Methods

### Data collection

The data in the present study were obtained from TCGA-Liver Hepatocellular Carcinoma. The data included RNA-seq expression of HN1 and clinical information of liver cancer patients with 50 normal liver tissue and 373 liver cancer tissue.

### Statistical analysis

Data management and analysis were performed using R version 3.5.2 [19]. The discrete difference of HN1 mRNA expression was visualized through boxplot by ggplot2 [20]. The pROC package evaluated the sensitivity and specificity of HN1 for the diagnosis of liver cancer [21]. The liver cancer patients were divided into two groups based on the optic value of HN1 mRNA expression by ROC [21]. We sought a correlation between HN1 expression and clinical features. Meanwhile, the combination of Kaplan–Meier and the log-rank test was used to compare the overall survival and relapse-free survival between the two groups from survival package [22]. Further, we used Univariate Cox analysis to screen for meaningful clinical variables, and assessed their prognostic value by Multivariate Cox analysis [22]. *P*<0.05 was considered a statistically significant difference.

### Gene Set Enrichment Analysis (GSEA)

Gene Set Enrichment Analysis (GSEA) determines whether an a priori defined set of genes has statistically significant differences in expression under two different biological conditions [23,24]. This analysis, performed using GSEA software 3.0 from the Broad Institute, was used for analysis of RNAseq data from TCGA-LIHC. The gene set of “h.all.v6.2.symbols.gmt”, which summarizes and represents specific, well-defined biological states or processes, was downloaded from the Molecular Signatures Database (http://software.broadinstitute.org/gsea/msigdb/index.jsp). The normalized enrichment score (NES) was determined by analysis of 1000 permutations. A gene set was considered significantly enriched when the *P*-value was less than 0.05 and the false discovery rate (FDR) was less than 0.25.

## Results

### Patient characteristic

Data including HN1 mRNA expression and basic clinical information had been collected from 373 liver cancer patients. The staging of TNM classification and stage was referred to AJCC [25]. Detailed data are shown in Table 1.

**Table 1:** The relationship between clinical features and of HN1 mRNA expression of liver cancer patients

Clinical characteristics	Variable	No. of patients	HN1 expression				χ2	*P*-value
			High	%	Low	%		
Age	<55	117	68	(37.16)	49	(25.93)	4.9326	**0.026**
	≥55	255	115	(62.84)	140	(74.07)		
Gender	Female	121	54	(29.35)	67	(35.45)	1.3177	0.251
	Male	252	130	(70.65)	122	(64.55)		
Histological type	Fibrolamellar carcinoma	3	1	(0.54)	2	(1.06)	0.4781	0.894
	Hepatocellular carcinoma	363	179	(97.28)	184	(97.35)		
	Hepatocholangiocarcinoma (mixed)	7	4	(2.17)	3	(1.59)		
Histologic grade	G1	55	21	(11.54)	34	(18.28)	11.4362	**0.009**
	G2	178	80	(43.96)	98	(52.69)		
	G3	123	72	(39.56)	51	(27.42)		
	G4	12	9	(4.95)	3	(1.61)		
Stage	I	172	67	(39.41)	105	(58.66)	14.0104	**0.002**
	II	87	49	(28.82)	38	(21.23)		
	III	85	52	(30.59)	33	(18.44)		
	IV	5	2	(1.18)	3	(1.68)		
T classification	T1	182	71	(38.59)	111	(59.36)	19.0295	**0.000**
	T2	95	56	(30.43)	39	(20.86)		
	T3	80	47	(25.54)	33	(17.65)		
	T4	13	10	(5.43)	3	(1.6)		
	TX	1	0	(0)	1	(0.53)		
N classification	N0	253	127	(69.4)	126	(66.67)	0.3334	0.840
	N1	4	2	(1.09)	2	(1.06)		
	NX	115	54	(29.51)	61	(32.28)		
M classification	M0	267	143	(77.72)	124	(65.61)	6.9333	**0.019**
	M1	4	2	(1.09)	2	(1.06)		
	MX	102	39	(21.2)	63	(33.33)		
Radiation therapy	No	340	165	(97.63)	175	(97.77)	0	1.000
	Yes	8	4	(2.37)	4	(2.23)		
Residual tumor	R0	326	153	(85.47)	173	(92.51)	5.6645	0.093
	R1	17	12	(6.7)	5	(2.67)		
	R2	1	1	(0.56)	0	(0)		
	RX	22	13	(7.26)	9	(4.81)		
Vital status	Deceased	130	84	(45.65)	46	(24.34)	17.7262	**0.000**
	Living	243	100	(54.35)	143	(75.66)		

### HN1 mRNA expression was up-regulated in patients with liver carcinoma

**Figure 1:**
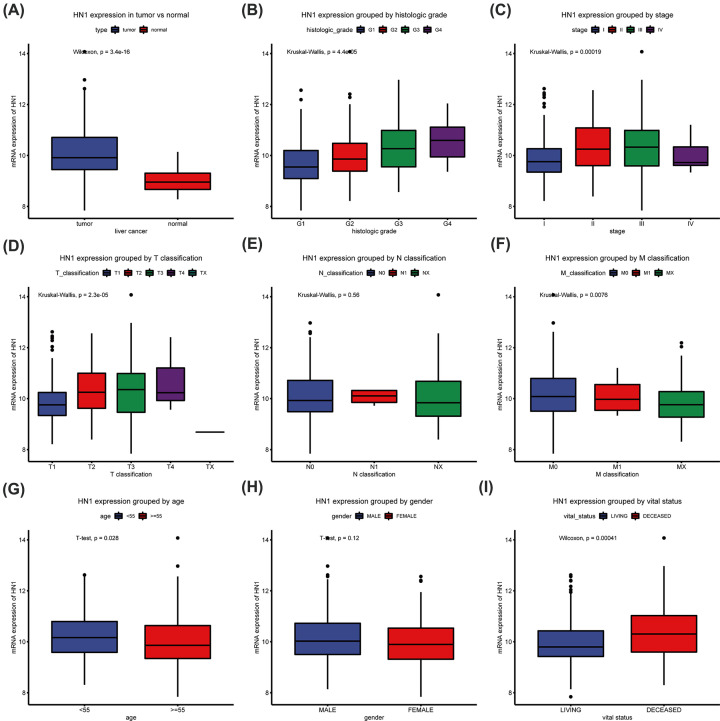
HN1 mRNA expression in liver cancer Differences of HN1 mRNA Expression in liver cancer (**A**) and grouped by different variables, such as histological grade (**B**), clinical stage (**C**), and TNM classification (**D–F**), age (**G**), gender (**H**), and survival status (**I**).

Box-plots showed that the HN1 mRNA expression in liver cancer patients was significantly higher than that in the normal group (*P*=3.4e^−16^) (Figure 1). In addition, HN1 expression also had significant differences in variable groups including histologic grade (*P*=4.4 e^−5^), clinical stage (*P*=0.00019), T classification (*P*=2.3 e^−5^), M classification (*P*=0.0076), age (*P*=0.028), and vital status (*P*=0.00041) (Figure 1). Especially, HN1 mRNA expression was increased with the malignant degree increased according to histologic grade.

### The diagnostic capability of HN1 in liver cancer

**Figure 2:**
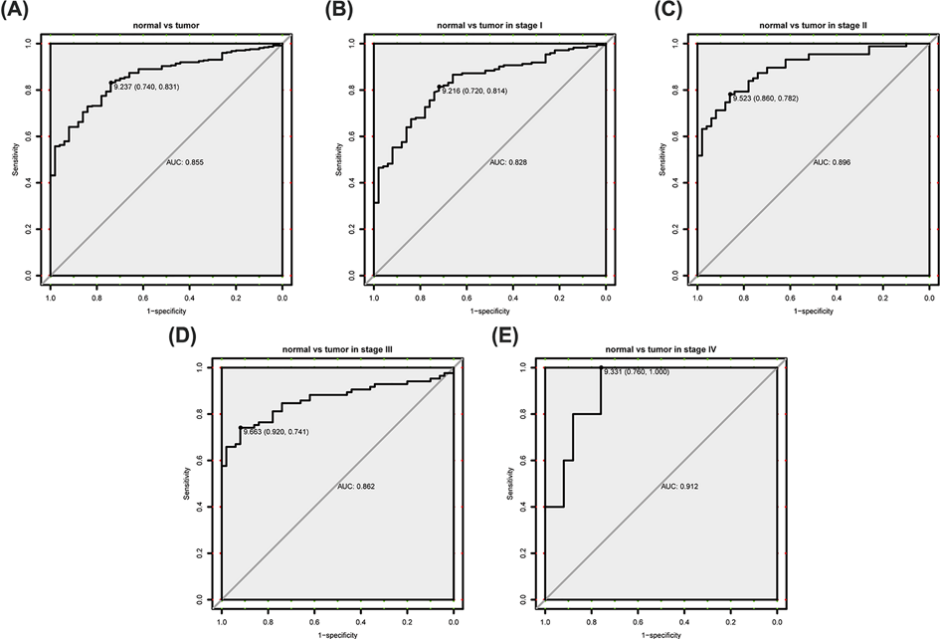
Diagnostic value of HN1 ROC of HN1 mRNA in liver cancer patients (**A**) and different clinical stages (**B–E**); Abbreviations: AUC, the area under the curve; ROC, receiver-operating characteristic curve.

As ROC curve showed, HN1 mRNA expression has ideal specificity and sensitivity for the diagnosis of all liver cancer patients (AUC = 0.855, Figure 2). As for different clinical stages, the diagnostic ability of HN1 also performed well in liver cancer (AUC of stages I–IV, 0.828,0.896,0.862,0.912) (Figure 2).

### Correlation between HN1 mRNA expression and clinical features of liver cancer

By comparing different clinical features in high and low expression groups of HN1 mRNA, we found the high expression of HN1 mRNA was associated with age (*P*=0.026), clinical stage (*P*=0.002), T classification (*P*<0.001), M classification (*P*=0.019), and vital status (*P*=0.000). With the deterioration of histologic grade, the frequency of high HN1 expression gradually increased (*P*=0.009) (Table 1).

### High HN1 predicts poor prognosis of OS and RFS

**Figure 3:**
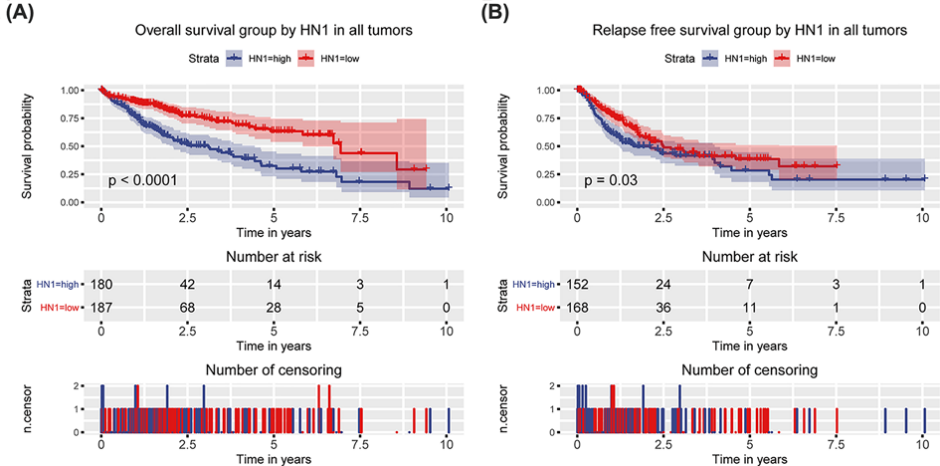
OS and RFS grouped by HN1 Differences of OS (**A**) and RFS (**B**) between HN1 low expression group and high expression group in liver cancer patients.

Kaplan–Meier survival curve with the log-rank test was performed to assess the prognostic value of HN1 in OS. The results showed that high HN1 expression was associated with poor OS (*P*<0.0001; Figure 3). Besides, survival curve with the log-rank test was performed to assess the prognostic value of HN1 in RFS. The results showed that high HN1 expression was associated with poor RFS (*P*=0.03; Figure 3).

### Subgroup analysis identified the prognostic value of HN1 in OS

**Figure 4:**
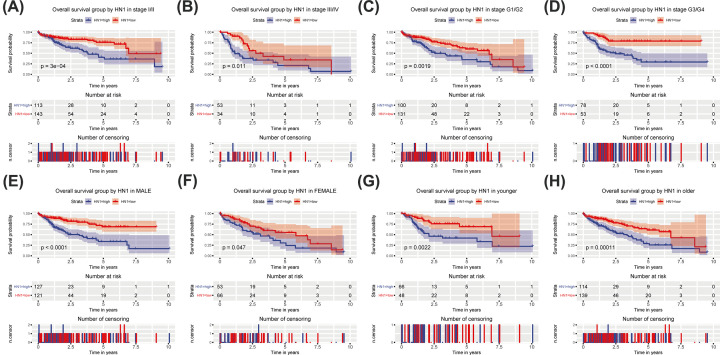
Subgroup analysis of OS Subgroup analysis of OS in specific cases, such as stage I/II (**A**), III/IV (**B**), histologic grade G1/G2 (**C**), G3/G4 (**D**), male (**E**), female (**F**), younger (**G**), and older (**H**).

Subgroup analysis showed that high HN1 expression was correlated with poor OS of cases with stage I/II (*P*=3e^−4^), stage III/IV (*P*=0.011), grade G1/G2 (*P*=0.0019), grade G3/G4 (*P*<0.0001), male (*P*<0.0001), female (*P*=0.047), younger (*P*=0.0022), and older (*P*=0.00011) (Figure 4).

### Subgroup analysis identified the prognostic value of HN1 in RFS

**Figure 5:**
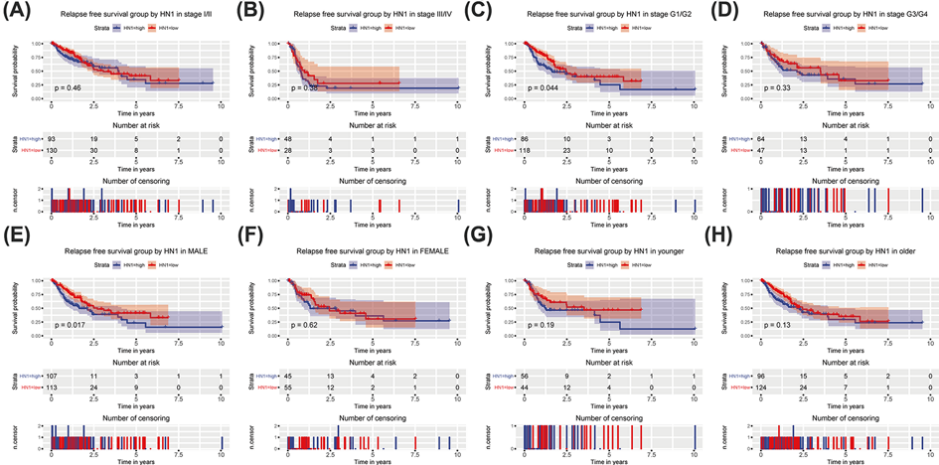
Subgroup analysis of RFS Subgroup analysis of RFS in specific cases, such as stage I/II (**A**), III/IV (**B**), histologic grade G1/G2 (**C**), G3/G4 (**D**), male (**E**), female (**F**), younger (**G**), and older (**H**).

Subgroup analysis showed that high HN1 expression was correlated with poor OS of cases with grade G1/G2 (*P*=0.044) and male (*P*=0.017) (Figure 5). However, stage I/II, stage III/IV, younger and older showed no significance in the prognostic value of HN1 in RFS.

### High HN1 is an independent prognostic factor for overall survival

**Figure 6:**
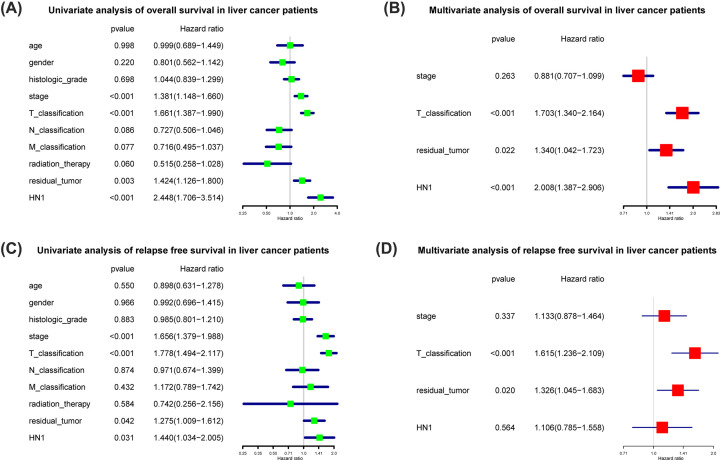
Univariate and multivariate cox analysis of OS and RFS in liver cancer patients Univariate (**A**) and multivariate (**B**) cox analysis of OS in liver cancer patients. Univariate (**C**) and multivariate (**D**) cox analysis of RFS in liver cancer patients.

Univariate cox analysis selected the potential OS related variables, including stage, T classification, residual tumor and HN1. Multivariate cox analyses showed that HN1 expression (HR = 2.008, 95% CI: 1.387–2.906, *P*<0.001), residual tumor (HR = 1.340, 95% CI: 1.042–1.723, *P*=0.022), T classification (HR = 1.703, 95% CI: 1.340–2.164, *P*<0.001) were independent risk factors for poor OS in liver cancer patients (Figure 6).

Further, we analyzed relapse-free survival in liver cancer patients. Univariate cox analysis selected the potential RFS-related variables, including stage, T classification, residual tumor and HN1. Multivariate cox analyses showed that residual tumor (HR = 1.326, 95% CI: 1.045–1.683, *P*=0.020), T classification (HR = 1.615, 95% CI: 1.236–2.109, *P*<0.001) were independent risk factors for poor RFS in liver cancer patients (Figure 6). However, HN1 showed no significance in multivariate analysis for relapse-free survival (*P*=0.564).

### GSEA identifies HN1-related signaling pathway

**Figure 7:**
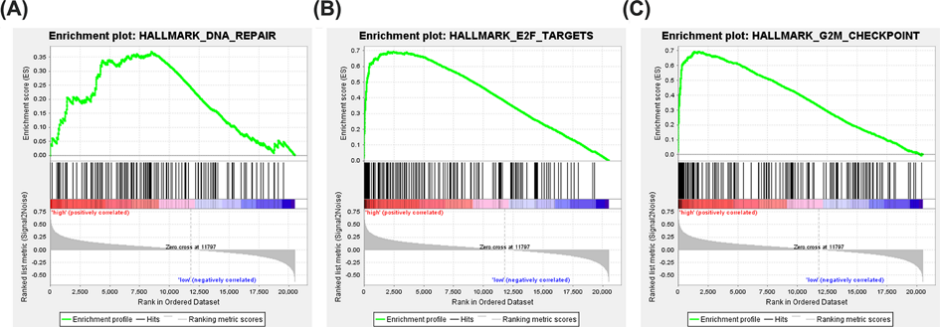
Signaling pathways activated in liver cancer patients with high HN1 phenotype Signaling pathways activated in liver cancer patients with high HN1 phenotype are DNA repair (**A**), E2F targets (**B**), and G2M checkpoint (**C**).

We compared the data sets for low and high HN1 expression using GSEA to identify signaling pathways activated during liver cancer. The results indicated significant differences (FDR < 0.25, NOM *P*-value < 0.05) in the enrichment of the MSigDB collection (h.all.v6.2.symbols.gmt; Table 2). We selected the most significantly enriched signaling pathways, based on normalized enrichment score (NES) (Figure 7, Table 2). The results indicated the data set with high HN1 expression was enriched for DNA repair, G2M checkpoint, E2F targets.

**Table 2:** Gene sets enriched in phenotype high

NAME	ES	NES	NOM *P*-val	FDR *q*-val
HALLMARK_DNA_REPAIR	0.370	1.817	0.013	0.222
HALLMARK_G2M_CHECKPOINT	0.694	1.779	0.012	0.147
HALLMARK_E2F_TARGETS	0.693	1.698	0.016	0.183

FDR: false discovery rate; NES: normalized enrichment score; NOM: nominal. Gene sets with NOM *P*-val <0.05 and FDR *q*-val <0.25 are considered as significant.

## Discussion

Liver cancer is one of the most intractable cancers with poor prognosis [1]. Finding promising biomarkers can accurately assess the prognosis and timely assist systemic treatment of liver cancer patients. Many researches have been seeking the diagnostic and prognostic biomarkers all the time [26–37]. Here, we first found that HN1 mRNA overexpressed in liver cancer. Meanwhile HN1 mRNA expression also plays a huge role in the diagnosis of liver cancer. In addition, by mining the clinical value of HN1 mRNA expression in different clinical variables, we found that the HN1 expression is associated with the histological grade, clinical stage, T classification, and M classification of liver cancer. Besides, HN1 mRNA expression predicted poor prognosis and is an independent prognostic factor for overall survival.

Previous studied has revealed HN1 mRNA overexpression in several cancers, such as prostate, lung, breast, epithelial ovarian cancer, and melanoma [13–18]. Consistent with these results, we found HN1 mRNA overexpression in liver cancer. This finding also makes it possible for HN1 to act as target molecules of liver cancer. HN1 can enhance oncogenic factor MYC [15], and the LEPR–STAT3 pathway, which manages the BCSC path and maintains CSC self-renewal [38–43]. MYC [15], LEPR, and STAT3 [16] signaling are all downstream regulators of HN1. The targeting HN1-treatment may simultaneously down-regulate three downstream cancer-promoting factors, and even block the signaling cascade of these beneficial cancers. Furthermore, the inhibition of HN1 may prevent the activation of compensation signals from three downstream cancer-promoting factors, to reduce the drug resistance of targeted drugs [44]. In addition, cell proliferation, invasion, migration, and metastasis of breast cancer are inhibited by targeting HN1 via miR-132 [45]. These indicate that HN1 may be an advantageous molecule for targeted therapy in liver cancer. Besides, the HN1 is an optimal diagnostic biomarker for liver cancer, due to our result that the HN1 has high specificity and sensitivity for the diagnosis.

Recent research shows that HN1 can promote the invasion of breast tumors by increasing the activity of MYC [15]. MYC is a famous oncogene which overexpresses in many tumors and plays a critical role in tumorigenesis and tumor proliferation, apoptosis [46]. This discovery is consistent with our results that the frequency of high expression of HN1 mRNA is associated with stage, histologic grade, T classification, and M classification. The up-regulation of HN1 expression may promote tumor growth and invasion, thereby it accelerates the progression of cancer and cause poor prognosis of liver cancer.

The poor prognosis of liver cancer is a worldwide serious challenge. Tumor recurrence and metastasis are important factors of poor prognosis. Cancer stem cells (CSCs) are closely related to tumor recurrence and metastasis. They can produce cancer cells that are different from themselves by differentiation and self-replicate to produce more stem cells. CSCs that survive under the treatment will initiate tumorigenesis [47]. Zhang et al. [15] pointed out that the HN1 overexpression promoted self-renewal of CSCs in breast. Another latest research reports that HN1 is identified as a critical regulatory gene for breast CSC maintenance and promotes breast cancer progression by LEPR-STAT3 pathway [16], which regularize CSC self-renewal [38–43]. In the study of melanoma and glioma, HN1 is also involved in the process of differentiation or dedifferentiation in cancer cells [10,17]. These suggest that HN1 may regulate the growth and differentiation of CSCs, promote the metastasis and recurrence of liver cancer, and lead to the poor prognosis of liver cancer patients finally. Consistent with these findings, we found that high HN1 could predict poor prognosis in liver cancer, which may involve in DNA repair, G2M checkpoint, and E2F targets.

In summary, we found HN1 mRNA overexpressed in liver cancer, explore the diagnostic value of HN1, and revealed that HN1 can independently predict and assess the prognosis of liver cancer patients. HN1 serves as a biomarker of diagnosis and prognosis in liver cancer. However, our study focuses on the clinical significance and do not explore the molecular mechanism. In future work, we will do some *in vivo* and *in vitro* experiments to explore the underlying mechanism in liver cancer.

## Abbreviations

CSC: cancer stem cell
GSEA: Gene Set Enrichment Analysis
HN1: hematological and neurological expressed 1
JPT1: Jupiter microtubule-associated homolog 1
NES: normalized enrichment score
OS: overall survival
RFS: recurrence-free survival
